# Isolation and Functional Analysis of *PISTILLATA* Homolog From *Magnolia wufengensis*

**DOI:** 10.3389/fpls.2018.01743

**Published:** 2018-11-26

**Authors:** Wen Liu, Xiangling Shen, Hongwei Liang, Yubing Wang, Zhengquan He, Dechun Zhang, Faju Chen

**Affiliations:** Key Laboratory of Three Gorges Regional Plant Genetics & Germplasm Enhancement (CTGU)/Biotechnology Research Center, China Three Gorges University, Yichang, China

**Keywords:** *PISTILLATA*, *Magnolia wufengensis*, ABC model, flower development, ectopic expression

## Abstract

*PISTILLATA* (*PI*) homologs are crucial regulators of flower development in angiosperms. In this study, we isolated the *MAwuPI* homolog from *Magnolia wufengensis*, a basal angiosperm belonging to the Magnoliaceae. Molecular phylogenetic analysis suggested that *MAwuPI* was grouped into the PI/GLO lineages of B-class MADS-box gene with the distinctive PI motif. Further expression profiling analysis showed that *MAwuPI* was expressed in tepals and stamens but not in juvenile leaves and carpels, similar to the spatial expression pattern of *AtPI* in Arabidopsis. Interestingly, *MAwuPI* had higher expression level in inner-tepals than in outer-tepals, whereas the *M. wufengensis* flower is homochlamydeous. Moreover, ectopic expression of *MAwuPI* in Arabidopsis *pi-1* mutant emerged filament-like structures but had no obvious petals, suggesting a partial phenotypic recovery of *pi-1* mutant. The features of *MAwuPI* in the expression pattern and gene function improved our acknowledgment of B-class genes in *M. wufengensis*, and contributed to the clarification of *M. wufengensis* evolution status and relations with other sibling species in molecular perspective.

## Introduction

Flowers, as the reproductive organ of plants, are the most complex structure of angiosperm (Endress, 2006). Compared to the reproductive structures of other plant groups, the flowers of angiosperm exhibit more variations in the number, morphology, size, color, and arrangement pattern of floral organs (Barrett, 2002). Thus, floral organ variation reflects angiosperm diversification. And the diversification of floral organ identity genes may have played a central role in the evolution of novel and different angiosperm morphologies (Irish, 2006).

The well-established ABCE model is widely accepted to explain the regular, sequential development of sepals, petals, stamens, and carpels in eudicot flowers (Bowman et al., 1991; Coen and Meyerowitz, 1991; Pelaz et al., 2000; Krizek and Fletcher, 2005). According to this model, the A-class genes determine the sepal and petal identity, the B-class genes specify petal and stamen identify, the C-class genes regulate stamen and carpel identity, and the E-class genes control the four whorl development. Moreover, the A-class and C-class genes function antagonistically. The combination of corresponding genes determines each floral organ identity. Although this model was built up on mutant studies in Arabidopsis and Antirrhinum, growing evidence showed that it may be suitable in all angiosperms (Theissen and Saedler, 2001; Kim et al., 2005; Melzer et al., 2010).

So far, most ABCE homeotic genes encode the MADS-box proteins, a huge transcriptional factor family (Becker and Theißen, 2003). MADS was named after its four major members, including MCM1, AGAMOUS, DEFICIENS, and SRF (Kim et al., 2004). Structural and molecular phylogenetic analysis indicated that plant MADS-box genes are belonging to MIKC-type MADS-box genes (Kaufmann et al., 2005), which contain four conserved domains, the MADS-, Intervening-, Keratin-like, and *C*-terminal (MIKC) domains (Becker and Theißen, 2003). The MADS domain, a highly conserved *N*-terminal domain, consists of about 58 amino acids (aa) and functions in dimerization by interaction with other transcriptional factors (Shore and Sharrocks, 1995). K domain, containing about 70 aa, is named because of the highly homologous primary structure with keratin. Three segments of α-helix (K1, K2, and K3) forms a coiled-coil secondary structure, involved in protein-protein interaction (Yang and Thomas, 2004). Between M domain and K domain lies the I domain (Krizek and Meyerowitz, 1996; Riechmann et al., 1996). The sequence and structure of the *C*-terminal region are highly variant, and play key roles in transcription factor activation, protein tetramerization, and functional specialization (Egea-Cortines et al., 1999; Honma and Goto, 2001; Lamb and Irish, 2003).

In Arabidopsis, *APETALA1* (*AP1*) and *AP2* belong to A-class genes, *PISTILLATA* (*PI*) and *AP3* belong to B-class genes, *AGAMOUS* (*AG*) and *AGAMOUS*-*LIKE* (*AGL*) belong to C-class genes, and *SEPALLATA1* (*SEP1*), *SEP2*, *SEP3*, and *SEP4* belong to E-class genes (Ditta et al., 2004; Krizek and Fletcher, 2005). Mutation in *AP2* gene results in the ectopic expression of C-class genes in outer two whorls, leading to sepal-to-carpel and petal-to-stamen homeotic transformation. On the contrary, in *ag* mutants, stamens converts into petals and another flower arises in place of the gynoecium (Bowman et al., 1991). Moreover, mutations in B-class genes convert the second whorl petal into a sepal structure and the third whorl stamen into a carpel structure (Goto and Meyerowitz, 1994). Recent researches further revealed that the ABC homeotic genes widely exist and are highly conserved among angiosperms (Becker and Theißen, 2003; Liu et al., 2010).

*Magnolia wufengensis* L.Y.Ma et L.R.Wang and *M. wufengensis var. multitepala* L.Y.Ma et L.R.Wang are newly identified species and variant, belonging to the Magnoliaceae, *Magnolia* (Ma et al., 2006). So far, *M. wufengensis* has only been found in mid-western Wufeng, Hubei Province in China, located in Wuling Mountain area, where many old plants originate and evolve. The limited distribution in wild and the local environmental disruption from human activities greatly restrict its regeneration in wild condition, and thus this species is critically endangered. However, the discovery of its wild population provides key information for identifying the center location of differentiation, distribution, and origination of modern Magnoliaceae family. Meanwhile, as a fine timber and landscape tree, as well as a precious medicinal tree, this species is also of great potential economic value. The flowers of *M. wufengensis* are highly different from core eudicots, like Arabidopsis. They have multiple morphologically identical petaloid tepals instead of distinct sepals and petals, and the numbers of the tepals vary from 9 to 24. In addition, the Manglietia plants as the relative early-diverging Magnoliaceae plants, have stable tepals, while the Michelia plants as the late-diverging Magnoliaceae plants, have largely varied tepals. And the Magnolia plants lies between the Manglietia and the Michelia plants during phylogenetic evolution. Thus, *M. wufengensis* L.Y.Ma et L.R.Wang and *M. wufengensis var*. *multitepala* L.Y.Ma et L.R.Wang provide ideal materials for the investigation on tepals development as well as the evolution and origin of the flowers in the Magnoliaceae plants.

B-class floral organ identity genes are key transcription factors controlling the development of petals and stamens (Kim et al., 2004). Previous studies have revealed that the diversity of flower organs in angiosperm is highly correlated with the variation of expression pattern and functional differentiation of B-class genes (Kim et al., 2004; Irish, 2006). Thus, phylogenetic and functional analysis of *M. wufengensis PISTILLATA* (*MAwuPI*) homolog may further reveal the mechanism of petal variation in *M. wufengensis* and clarify its evolution status in Magnolia plants.

## Materials and Methods

### Plant Material

Flower buds and juvenile leaves at different developmental stages were collected from *M. wufengensis* growing in the secondary natural forest at 1500 m elevation in Wufeng, southwest of Hubei Province. After sampled, flower buds were separated into outer-tepals, inner-tepals, stamens, and carpels (as shown in Supplementary Figure S1), and frozen immediately in liquid nitrogen.

The *pi-1* mutant was obtained by EMS (ethyl methanesulfonate) mutagenesis in the genetic background Ler (Goto and Meyerowitz, 1994), which contains a point mutation causing Trp80 (TGG) to stop code (TGA). The *pi-1* mutant and its corresponding wild-type Landsberg *erecta* were sown in soil and grown in controlled greenhouse at 22°C under 12 h light and 12 h dark cycles, with an irradiance of 120 μmol quanta m^−2^ s^−1^ and 70% relative humidity. Resistance selection and PCR-mediated genotyping were performed as Jing et al. (2015) previously described. The mature flowers of mutant and transgenic Arabidopsis plants were photographed by Leica165C stereomicroscope. For a better view, one of the petals has been removed before photographing.

### Isolation of *PI* Homolog in *M. wufengensis*

Total RNA of *M. wufengensis* were extracted using EASYspin plant RNA Extraction Kit (Aidlab, Beijing) according to the manufacturer’s instructions. And for each sample, 1 μg RNA was used for cDNA synthesis with RevertAid First Strand cDNA Synthesis Kit (Thermo Scientific, Waltham, MA, United Ststes) as previously described (Jing et al., 2014, 2015). A 659 bp 3′ end of *PI* homolog from *M. wufengensis* was amplified from the cDNA through 3′ RACE-specific primer GSPPI and 3′ RACE Outer Primer by the 3′-full RACE Core Set Ver.2.0 kit (TaKaRa, Japan), and a 910 bp 5′ end was amplified through 5′ RACE-specific primer PIGSP1 and PIGSP2 by 5′-full RACE Kit (TaKaRa, Japan). After assembly, the integrity of the cDNA sequences of the *PI* homolog from *M. wufengensis* were then verified using the forward primer MawuPIF and the reverse primer MawuPIR. PCR was performed with a 5 min at 94°C denaturation step, followed by 30 cycles of 45 s at 94°C, 45 s annealing at 56°C, 45 s extending at 72°C, with a final extension period of 10 min at 72°C. These PCR products were subcloned into pMD18-T Vector (TaKaRa, Japan) and sequenced. The primers used for isolation of *PI* homolog in *M. wufengensis* were listed in Supplementary Table S1.

### Sequence Alignments and Phylogenetic Analysis

Deduced amino acid sequences of MawuPI were blasted on the GenBank database. The molecular weight (MW) and theoretical isoelectric point (pI) of the coded protein were analyzed using the online ProtParam tool^1^ (Gasteiger et al., 2005). During the BLAST searches, multiple B class proteins from various angiosperm lineages were selected for alignment. Full-length amino acid sequences of these genes were aligned with a ClustalW program under default settings (Thompson et al., 1994) and phylogenetic trees were constructed by MEGA 5.0 software (Tamura et al., 2011) using the Neighbor-Joining (NJ) and Maximum Likelihood (ML) method with bootstrap setting as 1000 replicates. The accession numbers of the sequence used for phylogenetic analysis were listed in Supplementary Table S2.

### Semi-Quantitative and Quantitative RT-PCR

To analyze the expression level of *MawuPI* in different organs, total RNA was extracted from leaf, outer-tepals, inner-tepals, stamens and carpels of *M. wufengensis* as described above. Semi-quantitative PCR was performed as follows: 5 min at 94°C, 25 cycles of 30 s at 94°C, 30 s at 56°C, 30 s at 72°C, followed by 10 min at 72°C. PCR products was analyzed by electrophoresis in a 1% agarose gel and photographed under UV light. Then, quantitative RT-PCR was performed on IQ5 Multicolor Real-time PCR Detection System (Bio-Rad) to analyze the expression level of *MawuPI* in developmental stages. The reaction mixture was cycled as follows: 95°C for 3 min, followed by 40 cycles of 95°C for 10 s, 55°C for 20 s, 72°C for 20 s; the temperature changed by 0.5°C/s to 95°C for the melt curve. The *MAwuACTIN* gene was chosen as the internal control as previously reported (Wu et al., 2012). All experiments were performed with at least three biological replicates and three technical replicates. The gene specific primers used for semi-quantitative RT-PCR and quantitative RT-PCR were designed according to the isolated cDNA sequence. And the primer sequences were listed in Supplementary Table S1.

### Vector Construction and Arabidopsis Transformation

Full-length *MAwuPI* cDNA were amplified and cloned into a binary vector pBI121 (BD Biosciences Clontech) using *Xba*I and *Sma*I restriction enzymes. The *35S::MAwuPI* construction was transformed into heterozygous *pi-1* plant using the Agrobacterium-mediated floral-dip method (Clough and Bent, 1998). The transgenic Arabidopsis seeds were selected on a solid half-strength MS medium containing 50 μg/ml kanamycin at 4°C for 2 days, which were then transferred to the greenhouse for additional 10 days. Subsequently, the green seedlings were selected as positive seedlings and transplanted in soil. Then, genomic PCR and qRT-PCR were carried out to further confirm the *35S::MAwuPI* transgenic lines as previously described (Jing et al., 2015). Meanwhile, genotyping of wild-type, heterozygote and homozygous *pi-1* transformants was performed using a dCAPS marker (Neff et al., 2002; Jing et al., 2015). The primers used for vector construction and genotyping were listed in Supplementary Table S1.

## Results

### Cloning and Sequence Analysis of *MAwuPI*

To obtain the sequence of *MAwuPI*, homology-based cloning and RACE techniques were carried out to identify the full-length cDNA sequence. According to the assemble sequence, we further designed specific primers to amplify and verify the sequence. And we found that the cDNA of *MAwuPI* contains 1384 bp, including 573 bp of 5′-UTR, 172 bp of 3′-UTR, and 639 bp of ORF encoding for 212 amino acids. The molecular weight (MW) and isoelectric points (PI) of MAwuPI protein is 25 kD and 9.51, respectively. The sequence was deposited in GenBank under accession number JN315682.

**FIGURE 1:**
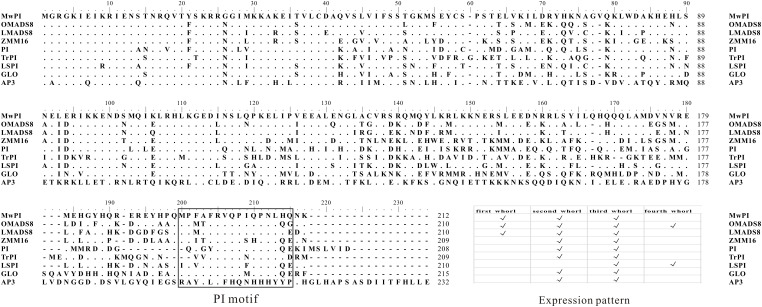
Comparison of deduced amino acid sequences encoded by MAwuPI and related members of the PI/GLO lineage. Dots indicate gaps inserted for alignment optimization. Sequence comparison of MAwuPI and the PI/GLO-related MADS domain proteins. The PI motif in the C-terminal region is boxed. The motif is highly conserved for PI/GLO lineage proteins with a DEF/AP3 protein (AtAP3) as a contrast. Meanwhile, a schematic expression of the selected genes in the four whorls of the flowers is also combined.

Further protein sequence comparisons were performed with seven PI/GLO proteins as well as a DEF/AP3 protein (AtAP3) as a contrast. Meanwhile, we have combined the phylogenetic analysis with a schematic expression in whorls of the selected genes (Figure 1). Our results showed that this protein contains a highly conserved MADS domain (1–59aa), a less conserved K domain (88–170aa), a least conserved I region (60–87aa) between M and K domain, and a highly conserved *PI* motif lying in *C*-terminal region (171–212aa) (Figure 1), which is essential for the formation of heterologous tetramer and functional specialization (Lamb and Irish, 2003; Kaufmann et al., 2005; Liu et al., 2010).

### Phylogenetic Analysis

**FIGURE 2:**
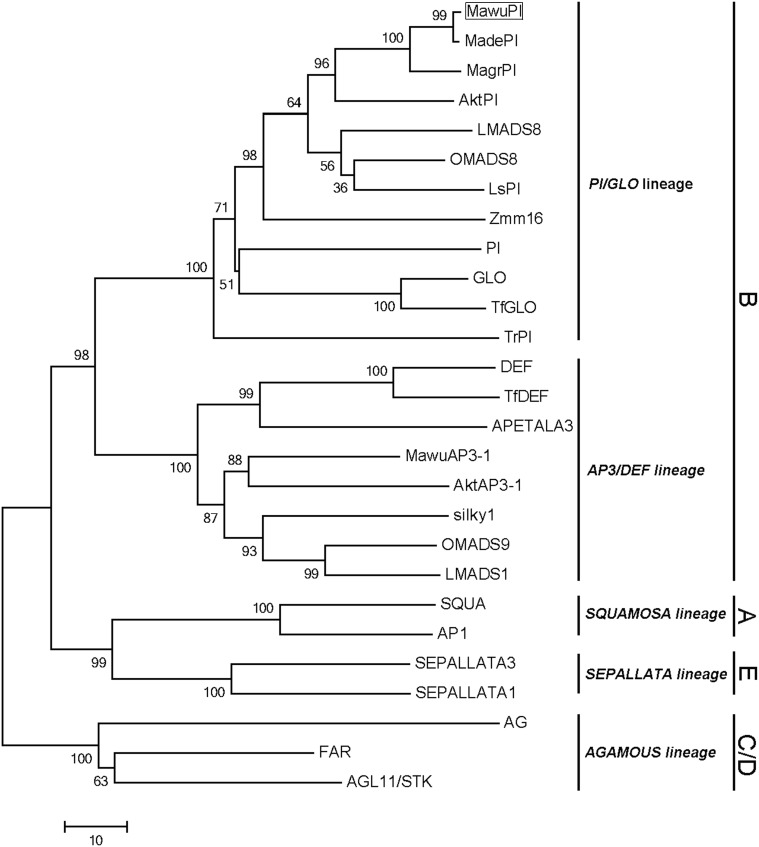
Phylogenetic analysis of PI/GLO lineage. The MAwuPI protein sequence is blasted with twenty B-class genes from other angiosperms, with two A-class genes, two C-class genes, one D-class gene and two E-class genes as outgroup. The MAwuPI is boxed.

To reveal the evolution of *MAwuPI*, the cloned *MAwuPI* sequence was then blasted with twenty B-class genes from other angiosperms (Supplementary Table S2). Meanwhile, two A-class genes, two B-class genes, one D-class gene, and two E-class genes were chosen as outgroup (Supplementary Table S2). Phylogenetic analysis suggested that MAwuPI is grouped into the PI/GLO lineages of B-class genes, together with other eleven PI/GLO lineage genes (Figure 2).

### Expression Profiling of *MAwuPI* in *M. wufengensis*

**FIGURE 3:**
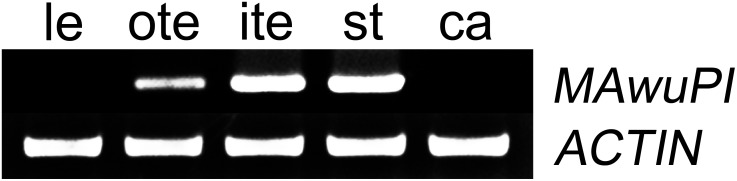
Semi-quantitative PCR analysis of the *MAwuPI* expression pattern in *Magnolia wufengensis*. For this assay, flower buds, and juvenile leaves were collected from *M. wufengensis* growing in the secondary natural forest. The flower buds were separated into outer-tepals, inner-tepals, stamens, and carpels, and frozen immediately in liquid nitrogen. Three biological replicates were performed for each sample. le, leaf; te, tepal; st, stamen; ca, carple; actin as internal control.

Since A-, B-, and C-class floral organ identity genes are expression in distinct floral whorls, we firstly analyzed the expression pattern of *MAwuPI* in different floral organs by semi-quantitative RT-PCR. As shown in Figure 3, *MAwuPI* was expressed in tepals and stamens, but not in juvenile leaves and carpels. This is similar to the spatial expression pattern of *AtPI* in Arabidopsis (Goto and Meyerowitz, 1994).

**FIGURE 4:**
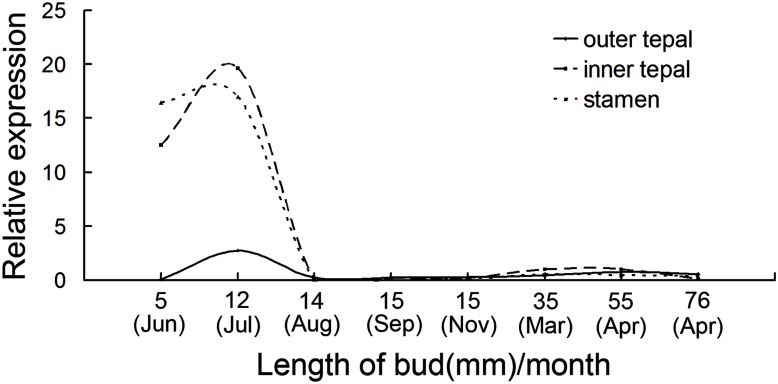
Expression pattern of *MAwuPI* at different developmental stages. For this assay, plant materials were collected from *M. wufengensis* growing in the secondary natural forest at different time-point, and frozen immediately in liquid nitrogen. Three biological replicates were performed at each time-point, and at least three technical replicates were performed for each data. Gene expression level was shown as fold changes in comparison to *MAwuactin*.

Next, quantitative RT-PCR was performed to monitor the dynamic changes of *MAwuPI* expression in out-tepals, inner-tepals, and stamens during different developmental stages. In June and July, when the primordium of inner-tepals and stamens initiated and grown fast, the expression of *MAwuPI* in inner-tepals and stamens elevated quickly and reached the peak in July when the flower buds were about 12 mm in length; in July and August, when the temperature continued to rise in southwest of Hubei province and the development of flower buds consequently slowed down, the expression of *MAwuPI* in inner-tepals and stamens decreased quickly (Figure 4). Although the expression of *MAwuPI* in out-tepals was comparatively lower, the variation trend matched those in inner-tepals and stamens (Figure 4). Additionally, from August onward, when the flower buds entered dormancy, the expression of *MAwuPI* in three whorls maintained in an extremely low level. Until March the following year, before flowering, its expression increased a little, but still largely lower than the peak (Figure 4).

### Ectopic Expression of *MAwuPI* Partially Rescues the Floral Deficiency of *pi-1* Mutant

**FIGURE 5:**
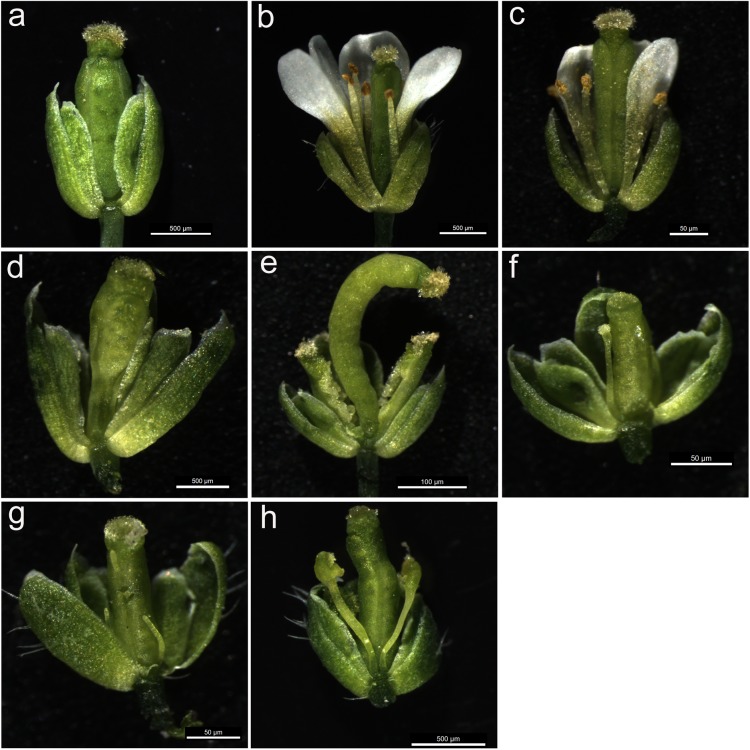
The mature flowers of transgenic plants ectopically expressing *MAwuPI* in Arabidopsis *pi-1* mutant. **(a)** The flower of *pi-1* homozygous *Arabidopsis thaliana*. **(b)** The flower of wild-type *Arabidopsis thaliana*. **(c)** The flower of *pi-1* heterozygous *Arabidopsis thaliana*. For a better view, one of the petals has been removed. **(d–h)** *35S::MAwuPI* transgenic Arabidopsis in *pi-1* mutant background (line 4^#^, 5^#^, 6^#^, 7^#^, and 8^#^, respectively).

**FIGURE 6:**
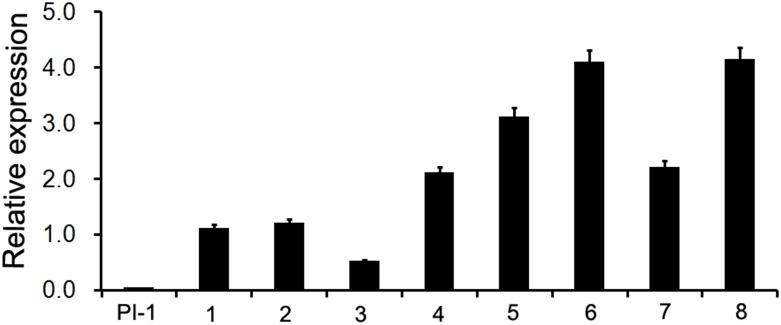
Quantitative Real-time PCR analysis of the *MAwuPI* transcript levels in complementary lines. At least three biological replicates and three technical replicates were performed for each data. Gene expression level was shown as fold changes, and the gene expression level in wild-type Arabidopsis plants was set as 1. Error bars represent standard error of the means of three biological repeats. *Atactin* is used as internal control. PI-1, *pi-1* mutant; 1–8, different *35S::MAwuPI* transgenic lines in Arabidopsis *pi-1* mutant background.

To further analyze the function of *MAwuPI*, complementation assay was performed by ectopic expression of *MAwuPI* into Arabidopsis *pi-1* mutant. The homozygous *pi-1* mutant (Figure 5a) is a strong allele expressing a truncated protein product of 79 aa, resulting in petals altered to sepals and stamens altered to carpels, while the heterozygote *pi-1* mutant (Figure 5c) exhibited a wild-type phenotype (Figure 5b; Goto and Meyerowitz, 1994). The transgenic plants were selected by kanamycin plates and PCR-mediated genotyping. A total of eight independent lines were obtained and assayed to monitor the expression levels of *MAwuPI* by qRT-PCR (Figure 6). Morphological observations showed that compared to *pi-1* mutant, five transgenic lines (4^#^, 5^#^, 6^#^, 7^#^, and 8^#^) ectopically expressing *MAwuPI* (Figures 5d–h) emerged filament-like structures in the third whorl of floral organ. This observation indicated that constitutive expression of *MAwuPI* can rescue the floral deficiency of *pi-1* mutant to different degrees. However, the second whorl was not obviously recovered (Figure 5), which implied that *MAwuPI* complemented the *pi-1* mutant only partially. Thus, these results suggested that *MAwuPI* plays a key role in stamen development.

## Discussion

In most core eudicots, *PISTILLATA* homologous genes, encoding the B-class transcription factors, are key regulators of petal and stamen development. In this study, we isolated and analyzed the expression patterns of *MAwuPI* during floral organ development and phylogenetic evolution between its closely related species. Meanwhile, we carried out functional complement experiments in Arabidopsis to further confirm its functions in specifying floral organ identities.

Though homology-based cloning and RACE techniques, we obtained the full-length cDNA sequence of *MAwuPI*. Molecular phylogenetic analysis suggested that *MAwuPI* belongs to the PI/GLO lineages of B-class MADS-box gene, since it contains the distinctive PI motif (Kim et al., 2004). Also, MAwuPI protein contains a highly conserved MADS domain, a less-conserved K domain, a least conserved I domain and a highly variant *C*-terminal domain. Thus, according to the sequence, *MAwuPI* is a typical B-class MADS-box gene.

The floral organ of a typical core eudicot composes of four whorls outside-to-inside, including sepals, petals, stamens, and carpels. In wild-type Arabidopsis, the expression of *PI* initiates after the formation of sepal primordium, which is firstly detected in the inner three whorls and later restricted in the second and third whorls of the floral organ. The silenced expression of *PI* in the fourth whorl during floral organ development might be due to the dependence between the expression of *PI* and *AP3*, which together confer B-class gene function (Jack et al., 1992; Goto and Meyerowitz, 1994; Krizek and Meyerowitz, 1996). Here, we found that *MAwuPI* was expressed in petaloid tepals and stamens (Figure 3), in accordance with the feature of B-class genes. Interestingly, the expression level of *MAwuPI* was dramatically higher in inner-tepals than in outer-tepals although the *M. wufengensis* flower is homochlamydeous. This expression pattern in *M. wufengensis* is different from that in Arabidopsis as well as other species (Figure 1). For instance, *LMADS8*, the *PI* homologous gene from *Lilium longiflorum*, was only expressed in stamens at early stage of flower bud differentiation, and then diffused to outer- and inner-tepals with equal expression level in these two whorls (Chen et al., 2012). In *Lacandonia schismatica*, the transcriptional activity of *LS-PI* was restricted in stamens and carpels but not in tepals, together with *LS-AP3* to regulate stamen development (Álvarez-Buylla et al., 2010). In *Oncidium* Gower Ramsey, the *PI* homologous gene *OMADS8* was expressed in vegetative organs and all whorls of floral organ, and regulated tepals differentiation in coordination with other MADS-box genes (Chang et al., 2010; Mao et al., 2015). In *Taihangia rupestris*, *TrPI* was expressed in both vegetative and reproductive organ, with top expression level in petals and stamens (Lu et al., 2010). In *Sesuvium portulacastrum*, *SpPI* was only expressed in stamens but not in tepals, and regulated the formation of stamen rather than tepals during floral organ development (Brockington et al., 2012). In basal eudicot *Eschscholzia californica*, *EScaGLO* was expressed in petals and stamens, modulating their development (Lange et al., 2013). Taken together, in most angiosperms, the *PI* homologs participate in regulating petals (petaloid tepals) and stamen development, while in a few cases (like *Lacandonia schismatica*) they are only involved in stamen formation. However, the expression of *PI* homologs is not limited in the tissues where they functions, indicating that the expression pattern and biological function of *PI* homologs undergo multiply evolutions during the origin and evolution of angiosperms.

In *M. wufengensis*, *MawuPI* was mainly expressed in inner-tepals and stamens (Figure 3). Functional complementation assay showed that ectopic expression of *MawuPI* in Arabidopsis *pi*-1 mutant emerged filament-like structures in the third whorl of floral organ, whereas petals were still defect (Figure 5). These observations indicated that *MawuPI* may mainly regulate stamens formation, but may not contribute to petal development or else may require other regulator(s). The *PI* homologs from gymnosperms have also been reported the similar phenotypes (Winter et al., 2002a; Wang et al., 2010), which supported the functional conservation of *PI* homologs during evolution. In addition, *LMADS8* was firstly expressed in stamens and then diffused to outer- and inner-tepals in monocotyledon *Lilium longiflorum*. And ectopic expression of *LMADS8* could significantly recover stamens defect and partially recover petals defect of *pi*-1 (Chen et al., 2012). Similar results were also observed in *Lacandonia schismatica* of the Triuridaceae (Álvarez-Buylla et al., 2010). Besides, in *Taihangia rupestris* of the Rosaceae, ectopic expression of *TrPI* could restore the phenotypic defects of both petals and stamens in Arabidopsis *pi*-1 mutant (Lu et al., 2010). Based on these data, we may conclude that during the evolution from gymnosperms to angiosperms, *PI* homologs were initially involved in stamens development, and then functioned in tepals development with the expression zone expanded.

Functional analyses have suggested that the involvement of *PI* homologs in controlling stamens development is quite conserved between species, but their roles in regulating petals development differ. In *Sesuvium portulacastrum* of the Aizoaceae, *SpPI* was only expressed in and regulated stamens (Brockington et al., 2012). In *Lacandonia schismatica* of the Triuridaceae, *LS-PI* was expressed in stamens and carpels but *LS-AP3* was only expressed in stamens, and the B-class genes mainly involved in specifying stamen identity (Álvarez-Buylla et al., 2010). Moreover, in most eudicots, *PI* homologs exert their function in modulating stamens and petals development, but their expression patterns in these species are largely varied (Lu et al., 2010; Sasaki et al., 2010; Lange et al., 2013; Fang et al., 2015). In general, their expression regions are much larger than where they exert their function. In other words, during evolution, floral organ identity genes expand their expression to new tissues, interact with local genes, and thereafter participate in organogenesis of these regions. In the Orchidaceae plants, as the group with most morphological diversity in monocotyledon, the tepals differentiation is highly correlated with the functions of B-class genes, which further supports this hypothesis (Chang et al., 2010; Mondragón-Palomino and Theissen, 2011). Interestingly, *MawuPI* has a higher expression level in stamens and inner-tepals than in outer-tepals, whereas the inner- and outer-tepals have no obvious morphological difference in *M. wufengensis*. However, heterodimeric complexes of DEF/AP3 and GLO/PI proteins are required for the development of tepals and stamens (Winter et al., 2002b). Thus, overexpressing only one of the two components might not be sufficient to recapitulate the function of *MAwuPI* in a wild-type context. On the other hand, although 35S promoter-drived complementation assay is widely used, regulatory elements necessary for correct *MAwuPI* expression are missing, which might affect the functions of *MAwuPI*. Thus, the mechanisms underlying *MAwuPI*-mediated tepals development remain to be further investigated.

Taken together, *MawuPI* was isolated and its functions in specifying floral organ identities were further characterized in this study. These data improved our knowledge of B-class genes in *M. wufengensis*, and contributed to the clarification of *M. wufengensis* evolution status and relations with other sibling species in molecular perspective.

## Author Contributions

FC conceived and directed this study. WL, XS, HL, and YW performed the experiments. WL and FC wrote the manuscript. WL, XS, ZH, and DZ analyzed the data and revised the manuscript. All authors approved the manuscript and the version to be published, and agreed to be accountable for all aspects of the work in ensuring that questions related to the accuracy or integrity of any part of the work are appropriately investigated and resolved.

## Conflict of Interest Statement

The authors declare that the research was conducted in the absence of any commercial or financial relationships that could be construed as a potential conflict of interest.

## Footnotes

1 http://web.expasy.org/protparam/

## Supplementary Material

FIGURE S1 Morphological observation of outer-tepals and inner-tepals in the flower of *M. wufengensis*.

TABLE S1 Primer sequences used in this study.

TABLE S2 Names and accession numbers of protein sequences used for phylogenetic analysis.
