# Supplementary figures and images for: Isolation and Functional Analysis of PISTILLATA Homolog From Magnolia wufengensis
Source: Front Plant Sci. 2018 Nov 26;9:1743. doi: 10.3389/fpls.2018.01743 (PMC6275295; doi:10.3389/fpls.2018.01743)

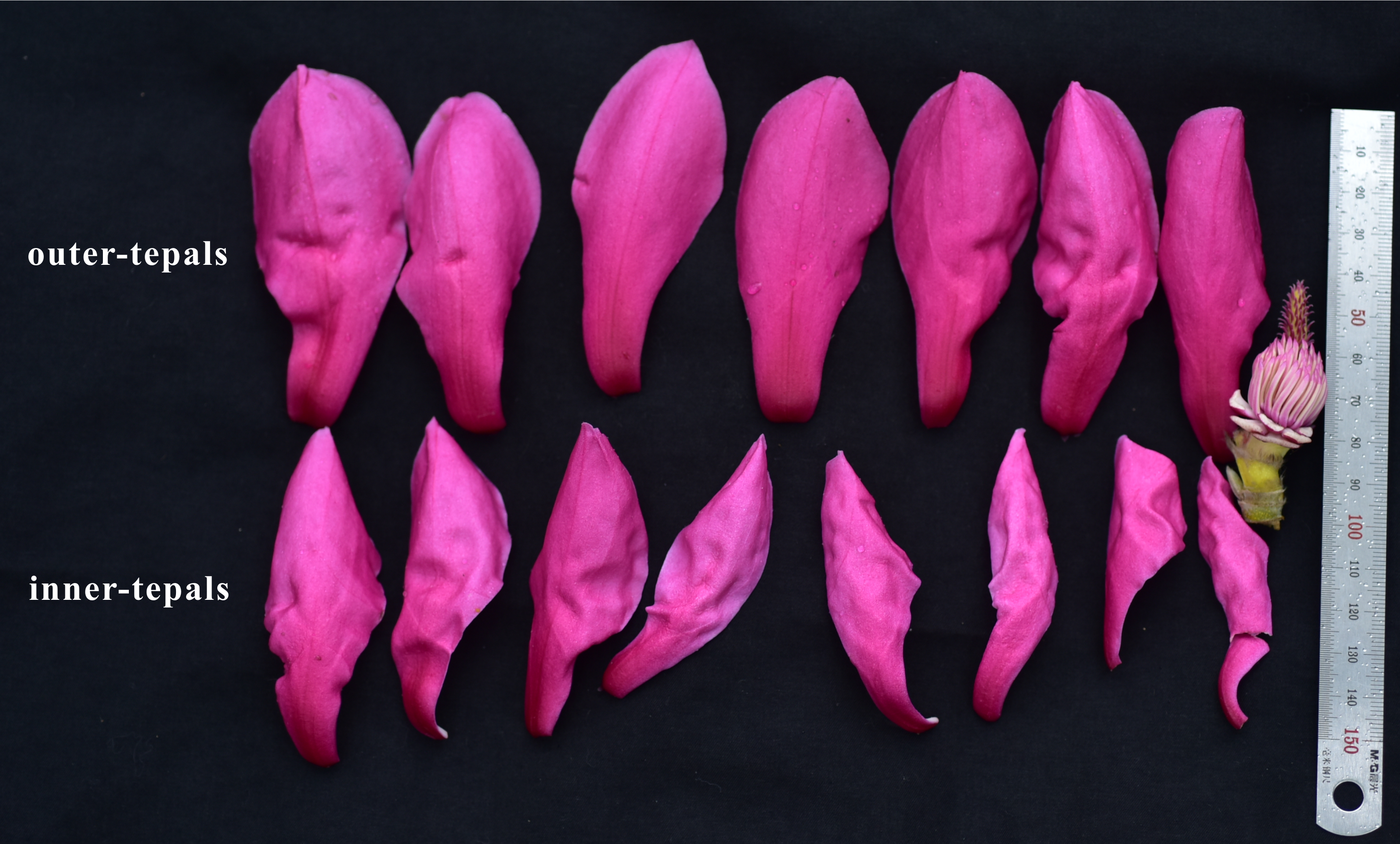

Supplement: FIGURE S1 — Morphological observation of outer-tepals and inner-tepals in the flower of M. wufengensis. [file Image_1.TIF]
