# Supplementary material for: Isolation and Functional Analysis of PISTILLATA Homolog From Magnolia wufengensis
Source: Front Plant Sci. 2018 Nov 26;9:1743. doi: 10.3389/fpls.2018.01743 (PMC6275295; doi:10.3389/fpls.2018.01743)
Supplement: TABLE S1 — Primer sequences used in this study. [file Table_1.DOCX]

**Table S1 Primer sequences used in this study.**

| **Primer name** | **Sequence** | **Usage** |
| --- | --- | --- |
| GSPPI | 5'-TGGGAAGATGTCCGAGTATTG-3' | 3′-RACE gene specific primer |
| 3′-race outer primer | 5'-TACCGTCGTTCCACTAGTGATTT3' | 3′-Race outer primer |
| PIGSP1 | 5'-GTAGATTGGGCTGGATGGGTTGC-3' | 5’-RACE gene specific primer |
| PIGSP2 | 5'-GCCTGTTATCCTCCTCCAGACTTC-3' | 5’-RACE gene specific primer |
| 5'-race outer primer | 5'-CATGGCTACATGCTGACAGCCTA-3' | 5’-RACE Outer primer |
| MawuPIF | 5'-TCTCTCTGCATCATCACCTCTCTC-3' | Verification of *MawuPI* sequence |
| MawuPIR | 5'-AGTTTTCCAAGCCATTGATGCCAG-3' | Verification of *MawuPI* sequence |
| RT-MawuPIF | 5'-CATCTCCATCTCTCTCTTCCTCGG-3' | Semi-quantitative RT-PCR |
| RT-MawuPIR | 5'-ATCGCCAGTTGTTGCTGATGCTGC-3' | Semi-quantitative RT-PCR |
| RT-MawuactinF | 5'-GCCGTGACCTGACAGATGCTCTTAT-3' | Semi-quantitative RT-PCR |
| RT-MawuactinR | 5'-CAGACTCGTCATACTCCGCCTTTG-3' | Semi-quantitative RT-PCR |
| qMawuPIF | 5'-TGAAGGGTGAGGATATCAACTC-3' | Quantitative RT-PCR |
| qMawuPIR | 5'-ATCGCCAGTTGTTGCTGATGCTGC-3' | Quantitative RT-PCR |
| qMAwuactinF | 5'-AAGAACATCCCGTCCTCCTTACTG-3' | Quantitative RT-PCR |
| qMAwuactinR | 5'-ACCGGAATCAAGCACAATACCTGT-3' | Quantitative RT-PCR |
| TMawuPIF | 5'-CTCTAGATCATCACCTCTCTCCATC-3' | Vector construction |
| TMawuPIR | 5'-GAGCTCTTGATGCCAGAGCATCCGTTC-3' | Vector construction |
| TatPI-F | 5'-TACCAGAAGTTATCTGGCAAGAAATCATG-3' | Genotyping of *pi-1* |
| TatPI-R | 5'-CCAATTTCATGATATCTAGCTCAG-3' | Genotyping of *pi-1* |
| qAtactinF | 5'-CGTATGAGCAAGGAGATCAC-3' | Quantitative RT-PCR |
| qAtactinR | 5'-CACATCTGTTGGAAGGTGCT-3' | Quantitative RT-PCR |
