# Supplementary material for: Isolation and Functional Analysis of PISTILLATA Homolog From Magnolia wufengensis
Source: Front Plant Sci. 2018 Nov 26;9:1743. doi: 10.3389/fpls.2018.01743 (PMC6275295; doi:10.3389/fpls.2018.01743)
Supplement: TABLE S2 — Names and accession numbers of protein sequences used for phylogenetic analysis. [file Table_2.DOCX]

**Table S2 Names and accession numbers of protein sequences used for phylogenetic analysis.**

| **Name** | **Species** | **Accession number** | **Protein type** |
| --- | --- | --- | --- |
| MawuPI | *Magnolia wufengensis* | AFM75882 | PI/GLO lineage（B-class） |
| OMADS8 | *Oncidium* Gower Ramsey | ADJ67236 | PI/GLO lineage（B-class） |
| LMADS8 | *Lilium longiflorum* | AEI88009 | PI/GLO lineage（B-class） |
| Zmm16 | *Zea mays* | NP_001105136 | PI/GLO lineage（B-class） |
| PI | *Arabidopsis thaliana* | AAD51996 | PI/GLO lineage（B-class） |
| TrPI | *Taihangia rupestris* | ABB59993 | PI/GLO lineage（B-class） |
| LSPI | *Lacandonia schismatica* | ADC53258 | PI/GLO lineage（B-class） |
| GLO | *Antirrhinum majus* | CAA48725 | PI/GLO lineage（B-class） |
| MadePI | *Magnolia denudata* | AFN68764 | PI/GLO lineage（B-class） |
| MagrPI | *Magnolia grandiflora* | AFN68766 | PI/GLO lineage（B-class） |
| AktPI | *Akebia trifoliate* | AAT46101 | PI/GLO lineage（B-class） |
| TfGLO | *Torenia fournieri* | BAJ15423 | PI/GLO lineage（B-class） |
| silky1 | *Zea mays* | NP_001104951 | AP3/DEF lineage（B-class） |
| MAwuAP3_1 | *Magnolia wufengensis* | AFM75880 | AP3/DEF lineage（B-class） |
| OMADS9 | *Oncidium* Gower Ramsey | ADJ67235 | AP3/DEF lineage（B-class） |
| LMADS1 | *Lilium longiflorum* | AAM27456 | AP3/DEF lineage（B-class） |
| AktAP3-1 | *Akebia trifoliate* | AAT46097 | AP3/DEF lineage（B-class） |
| DEF | *Antirrhinum majus* | CAA44629 | AP3/DEF lineage（B-class） |
| APETALA3 | *Arabidopsis thaliana* | AEE79216 | AP3/DEF lineage（B-class） |
| TfDEF | *Torenia fournieri* | BAG24492 | AP3/DEF lineage（B-class） |
| SQUA | *Antirrhinum majus* | CAA45228 | SQUAMOSA lineage（A-class） |
| AP1 | *Arabidopsis thaliana* | CAA78909 | SQUAMOSA lineage（A-class） |
| FAR | *Antirrhinum majus* | CAB42988 | AGAMOUS lineage（C-class） |
| AG | *Arabidopsis thaliana* | AEE84112 | AGAMOUS lineage（C-class） |
| AGL11/STK | *Arabidopsis thaliana* | AEE82818 | AGAMOUS lineage（D-class） |
| SEPALLATA1 | Arabidopsis thaliana | AAU81996 | SEPALLATA lineage（E-class） |
| SEPALLATA3 | *Arabidopsis thaliana* | AEE30503 | SEPALLATA lineage（E-class） |
